# Barrett’s Metaplasia Progression towards Esophageal Adenocarcinoma: An Attempt to Select a Panel of Molecular Sensors and to Reflect Clinical Alterations by Experimental Models

**DOI:** 10.3390/ijms23063312

**Published:** 2022-03-18

**Authors:** Edyta Korbut, Kinga Krukowska, Marcin Magierowski

**Affiliations:** Cellular Engineering and Isotope Diagnostics Laboratory, Department of Physiology, Jagiellonian University Medical College, 16 Grzegorzecka Street, 31-531 Cracow, Poland; edyta.korbut@uj.edu.pl (E.K.); kinga.krukowska@doctoral.uj.edu.pl (K.K.)

**Keywords:** gastroesophageal reflux disease, Barrett’s esophagus, esophageal adenocarcinoma, bile acids, translational model, cancer transformation

## Abstract

The molecular processes that predispose the development of Barrett’s esophagus (BE) towards esophageal adenocarcinoma (EAC) induced by gastrointestinal reflux disease (GERD) are still under investigation. In this study, based on a scientific literature screening and an analysis of clinical datasets, we selected a panel of 20 genes covering BE- and EAC-specific molecular markers (*FZD5*, *IFNGR1*, *IL1A, IL1B, IL1R1, IL1RN, KRT4, KRT8, KRT15, KRT18, NFKBIL1, PTGS1, PTGS2, SOCS3, SOX4, SOX9, SOX15, TIMP1, TMEM2, TNFRSF10B*). Furthermore, we aimed to reflect these alterations within an experimental and translational in vitro model of BE to EAC progression. We performed a comparison between expression profiles in GSE clinical databases with an in vitro model of GERD involving a BE cell line (BAR-T) and EAC cell lines (OE33 and OE19). Molecular responses of cells treated with acidified bile mixture (BM) at concentration of 100 and 250 μM for 30 min per day were evaluated. We also determined a basal mRNA expression within untreated, wild type cell lines on subsequent stages of BE and EAC development. We observed that an appropriately optimized in vitro model based on the combination of BAR-T, OE33 and OE19 cell lines reflects in 65% and more the clinical molecular alterations observed during BE and EAC development. We also confirmed previous observations that exposure to BM (GERD in vitro) activated carcinogenesis in non-dysplastic cells, inducing molecular alternations in the advanced stages of BE. We conclude that it is possible to induce, to a high extent, the molecular profile observed clinically within appropriately and carefully optimized experimental models, triggering EAC development. This experimental scheme and molecular marker panel might be implemented in further research, e.g., aiming to develop and evaluate novel compounds and prodrugs targeting GERD as well as BE and EAC prevention and treatment.

## 1. Introduction

Esophageal cancer (EC) is a pathological status of esophageal mucosa with a high mortality rate due to its aggressive course, poor prognosis and the late stage of most diagnoses. EC ranks sixth in terms of mortality overall, with an average 5-year survival of 18.4%, making EC a leading cause of deaths worldwide [1,2,3]. Histologically, the primary esophageal cancers are divided into two subtypes: squamous cell carcinoma (ESCC) and esophageal adenocarcinoma (EAC), which is derived from Barrett’s esophagus (BE) [4]. ESCC and EAC have different disease etiology, risk factors, incidence trends and locations in the esophagus. Whilst the incidence of ESCC has been decreasing in some areas of the world due to improved living conditions and endoscopic screening programmes, there has been a dramatic increase in the incidence of EAC in developed countries over the past four decades [4,5,6].

The mechanisms of progression from dysplasia to an invasive ESCC has not been well established; however, epidemiologic studies have indicated that recurrent physicochemical insult to the esophageal mucosa, including tobacco smoking and alcohol abuse, increase the risk of cancer [4,7]. Moreover, several studies showed that many hereditary factors and gene mutations (*TP53, NOTCH1, PI3KCA, CDKN2A, KMT2D, PTEN*) are involved in the etiology of ESCC [4,8,9]. In contrast, EAC is a carcinoma with a glandular structure preceded by BE [10]. BE is a complex, premalignant condition characterized as a replacement of the esophageal squamous epithelium by an intestinal-type columnar epithelium with a crypt-like architecture [11,12]. It has been confirmed that BE, as well as EAC, are strongly associated with gastroesophageal reflux disease (GERD), a chronic regurgitation of gastric contents into the lower esophagus [13,14]. The gastric refluxate contains gastric secretions (hydrochloric acid and pepsin) as well as alkaline duodenal contents (bile salts), leading to mucosal damage [10,13,15]. Moreover, it is estimated that approximately 10–30% of well-developed countries’ societies are susceptible to GERD [16]. Among various factors, age, male sex, white race, smoking, alcohol consumption and obesity are considered major risk factors for GERD [17]. Depending on the dysplasia type, approximately 0.3% of annual BE cases transform to EAC [18].

A growing body of evidence has shown that progression of BE to EAC is a multi-step process that involves the development of non-dysplastic BE to low-grade dysplasia (LGD), followed by its progression to high-grade dysplasia (HGD) and ultimately to EAC [19,20]. Nevertheless, the molecular mechanisms underlying the development of BE and its progression to EAC are largely unknown.

Deeply evaluated molecular mechanisms underlying the generation of bile mixture (BM)-induced esophageal lesions may contribute to the development of innovative pro-drugs and pharmacological treatment methods. Nonetheless, recent studies have undertaken an approach to set up an in vitro model of BE since the available translational animal models are relatively difficult to handle and require appropriate surgical skills or specific infrastructure [21,22,23]. Various cell cultures have been studied to investigate bile and acid reflux and its role in stimulation and/or inhibition of BE development [24]. Moreover, exposure of esophageal mucosa or esophageal cells to BM was suggested previously to be linked with further BE progression and EAC development [25,26,27].

Our previous work showed that the treatment of a primary immortalized human esophageal epithelial squamous cell line (EPC2) and human normal esophageal epithelial cell line (HET-1A) with acidified medium (pH 5.0) and BM altered gene expression, reflecting a clinical human BE-specific gene expression profile [21]. For example, an analysis of the expression profile of keratins (KRTs), a major constituent of the esophageal epithelium, revealed significant changes from squamous epithelia specific towards those expressed in columnar epithelium, as observed within the analysis of clinical samples [21]. However, it is unclear whether the molecular changes in BE- or EAC-derived cell lines and the previously implemented experimental design will also reflect the dysplastic progression of BE to adenocarcinoma.

Therefore, we aimed to select a panel of 20 targets possibly changed in BE and EAC based on a scientific literature screen and the further confirmation of these alterations in clinical biopsies. Next, we assessed how the selected markers were expressed on subsequent stages of esophageal metaplasia development and progression reflected by non-neoplastic, telomerase-immortalized Barrett’s epithelial cells (BAR-T) and two EAC cell lines (OE33 and OE19). Furthermore, we tested whether the expression of selected genes might be changed upon chronic exposure of each cell line to various concentrations of acidified BM, aiming to establish an optimized experimental GERD model altering the selected sensors.

## 2. Results

### 2.1. Evaluation of the Molecular Profile of EAC and BE in Clinical Samples According to the GSE Datasets

Table 1 shows that in EAC biopsies, the mRNA expression of *FZD5, IFNGR1, SOCS3, TIMP1, TNFRSF10B, IL1B, TMEM2, KRT8, KRT18, SOX4, SOX9* and *PTGS2* was significantly upregulated compared to normal squamous epithelium (*p* < 0.05, logFc > 1). The *mRNA* expression of *IL1RN, IL1A, NFKBIL1, KRT4, KRT15, SOX15* and *PTGS1* was significantly decreased in EAC samples compared with squamous epithelium (*p* < 0.05, logFc < −1, Table 1). Alterations of *IL1R1 mRNA* expression did not meet the criteria of statistical and biological significance (Table 1). In EAC biopsies, the mRNA expression of *IL1RN, IL1A, KRT4, KRT15, SOX15* and *PTGS1* was significantly decreased compared to BE biopsies (*p* < 0.05, logFC < −1, Table 1). The mRNA expression of *IFNGR1, SOCS3, TIMP1, TNFRSF10B, IL1B, IL1R1, NFKBIL1, TMEM2, KRT8, KRT18, SOX4, SOX9, FZD5* and *PTGS2* did not meet the selected criteria (Table 1).

Table 2 shows that in BE samples, the mRNA expression of *IFNGR1, SOCS3, TIMP1, TNFRSF10B, TMEM2, IL1B, FZD5, SOX4, SOX9, PTGS2* was significantly increased (*p* < 0.05, logFc > 1) in at least one out of three datasets, whereas mRNA expression of *IL1RN, IL1A, NFKBIL1, SOX15* and *PTGS1* was significantly decreased in comparison to squamous epithelium (*p* < 0.05, logFC < −1). The gene expression of *IL1R1* did not fulfill the criteria (Table 2). The mRNA expression of *KRT4, KRT15* was significantly downregulated in BE samples, whereas mRNA expression of *KRT8, KRT18* was significantly upregulated in BE samples compared to normal epithelium and based on the analysis published elsewhere [21].

### 2.2. Molecular Profile of Normal Esophageal Epithelium, BE and EAC Representing Cell Lines

**Figure 1:**
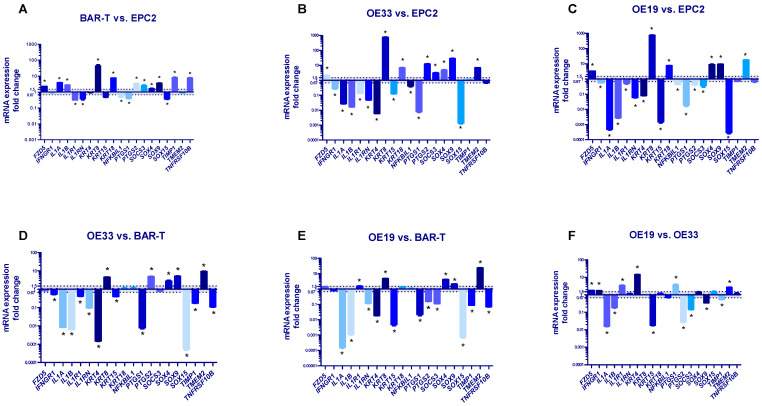
Alterations in molecular profile of BAR-T, OE33 and OE19 cells representing subsequent stages of Barrett’s esophagus (BE) and esophageal adenocarcinoma (EAC) development. (**A**–**C**): mRNA fold change for selected genes was normalized to the basal expression for EPC2 cell line; (**D**,**E**): results are normalized to the basal expression for BAR-T cells; (**F**): mRNA expressions in OE19 are normalized to the values observed in OE33 cells. Dotted lines indicate 1.5-fold up-/downregulation of mRNA expression compared to the reference cell line. Results shown as the mean ± SEM of 3 values per group for each gene. Statistically and biologically significant differences compared to reference cell line are indicated by asterisk (*) (*p* < 0.05).

Figure 1A shows significantly increased mRNA expression of *FZD5, IL1A, IL1B, KRT8, KRT18, PTGS2, SOCS3, SOX4, SOX9, TIMP1* and *TNFRSF10B,* significantly decreased mRNA expression of *IL1R1, IL1RN, NFKBIL1, PTGS1, SOX15* (*p* < 0.05) and no change in mRNA expression of *IFNGR1, KRT15, KRT4, TMEM2* in BAR-T cells in reference to EPC2 cells. The mRNA expression of *FZD5, KRT8, KRT18, PTGS2, SOCS3, SOX4, SOX9* and *TMEM2* (but not *TIMP1*) was significantly upregulated in OE33 cells in comparison to EPC2 cells (*p* < 0.05, Figure 1B). The mRNA expression of *IFNGR1, IL1A, IL1B, IL1R1, IL1RN, KRT4, KRT15, NFKBIL1, PTGS1* and *SOX15* but not *TNFRSF10B* was significantly decreased in OE33 compared to EPC2 (*p* < 0.05, Figure 1B). In OE19 cells, mRNA expression of *FZD5, KRT8, KRT18, SOX4, SOX9* and *TMEM2* was significantly elevated, while mRNA expression of *IFNGR1*, *IL1A, IL1B, IL1R1, IL1RN, KRT4, KRT15, NFKBIL1, PTGS1, PTGS2, SOCS3* and *SOX15* was significantly downregulated in comparison to EPC2 cells (*p* < 0.05, Figure 1C). There were no statistically significant changes in mRNA expression of *TIMP1* and *TNFRSF10B* between OE19 and EPC2 cell lines (Figure 1C).

Figure 1D shows significantly increased mRNA expression of *KRT8*, *PTSG2, SOX4, SOX9, TMEM2* and decreased mRNA expression of *IFNGR1, IL1A, IL1B, IL1R1, IL1RN, KRT4, KRT15, PTGS1*, *SOX15, TIMP1* and *TNFRSF10B* in OE33 cells in reference to BAR-T cells (*p* < 0.05, Figure 1D). *FZD5, KRT18*, *NFKBIL, SOCS3* mRNA expression was not significantly changed (*p* < 0.05, Figure 1D). In OE19 cells, mRNA expression of *IL1R1, KRT8, SOX4, SOX9* and *TMEM2* was significantly upregulated, whereas *IL1A, IL1B, IL1RN, KRT4, KRT15, PTGS1, PTGS2*, *SOCS3, SOX15, TIMP1, TNFRSF10B* were significantly downregulated in comparison to BAR-T cells (*p* < 0.05, Figure 1E). There was no statistically significant change in *FZD5, IFNGR1, KRT18* and *NFKBIL1* mRNA expression between OE19 and BAR-T cell lines (*p* < 0.05, Figure 1E).

Figure 1F shows that in OE19 cells, mRNA expression of *FZD5, IFNGR1, IL1R1, KRT4, PTGS1* and *TMEM2* was significantly upregulated, whereas *IL1A, IL1B, KRT15, PTGS2, SOCS3, SOX9* and *TIMP1* mRNA expression was significantly downregulated compared to OE33 (*p* < 0.05, Figure 1F). *IL1RN, KRT8, KRT18, NFKBIL1, SOX4, SOX15* and *TNFRSF10B* mRNA expression was not significantly different between OE19 and OE33 cells (Figure 1F).

### 2.3. Cells Viability and the Resistance to Various Concentrations of BM on Subsequent Stages of Esophageal Metaplasia Development

**Figure 2:**
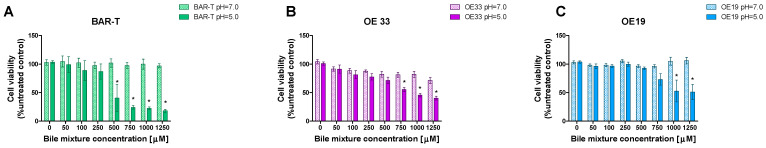
The viability of cells in different developmental stages of Barrett’s esophagus (BE) and esophageal adenocarcinoma (EAC) exposed to various concentrations of bile mixture (BM) applied in pH 5.0 and pH 7.0. BAR-T (**A**), OE33 (**B**) and OE19 (**C**) were treated for 30 min with 50–1250 μM of BM. Results are shown as the mean ± SEM. Asterisk (*) indicates a statistically significant difference as compared with respective BM concentration applied in pH 5.0 (*p* < 0.05).

The viability (%) of BAR-T cells was significantly decreased in pH 5.0 compared to pH 7.0 when BM was applied in concentrations of 500 μM and higher but not in 250 μM and lower (*p* < 0.05, Figure 2A). The viability (%) of OE33 cells was significantly decreased in pH 5.0 compared to pH 7.0 when BM was applied in concentrations of 750 μM and higher but not in 500 μM and lower (*p* < 0.05, Figure 2B). The viability (%) of OE19 cells was significantly decreased in pH 5.0 compared to pH 7.0 when BM was applied in concentrations of 1000 μM and higher but not in 750 μM and lower (*p* < 0.05, Figure 2B).

### 2.4. The Effect of Chronic GERD on the Molecular Profile of Cells Representing Subsequent Stages of Esophageal Metaplasia and Esophageal Adenocarcinoma

**Figure 3:**
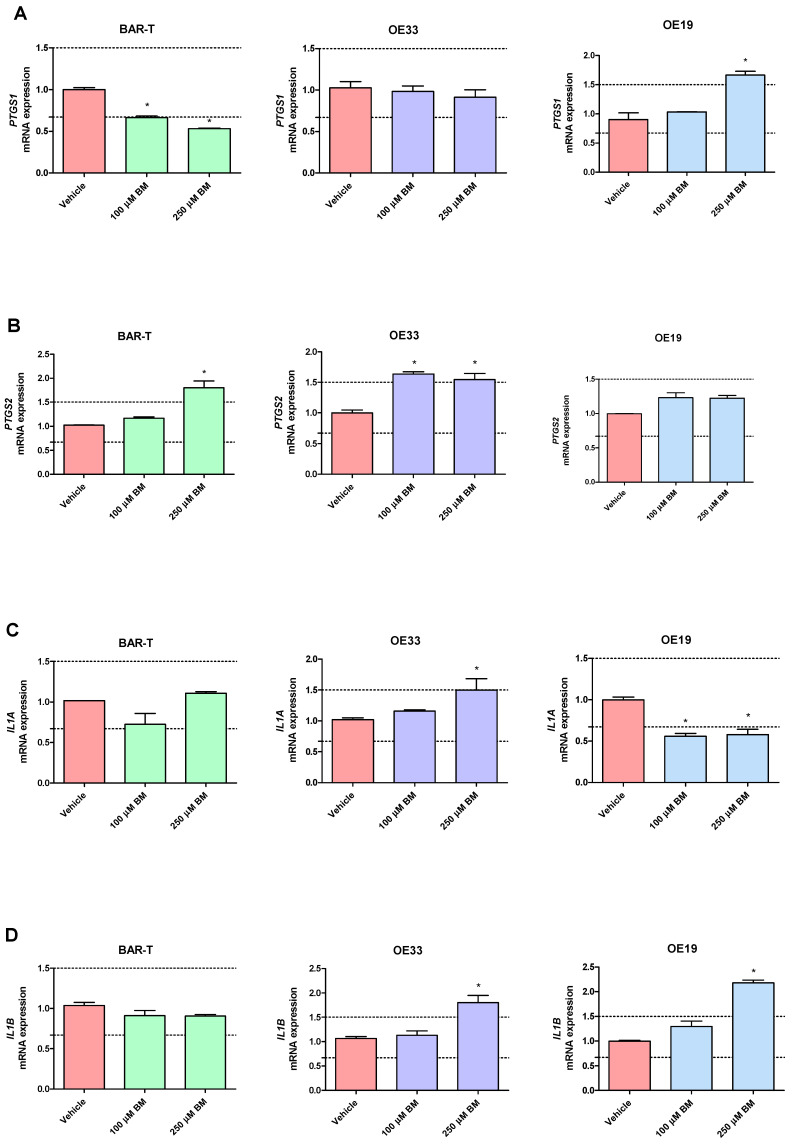

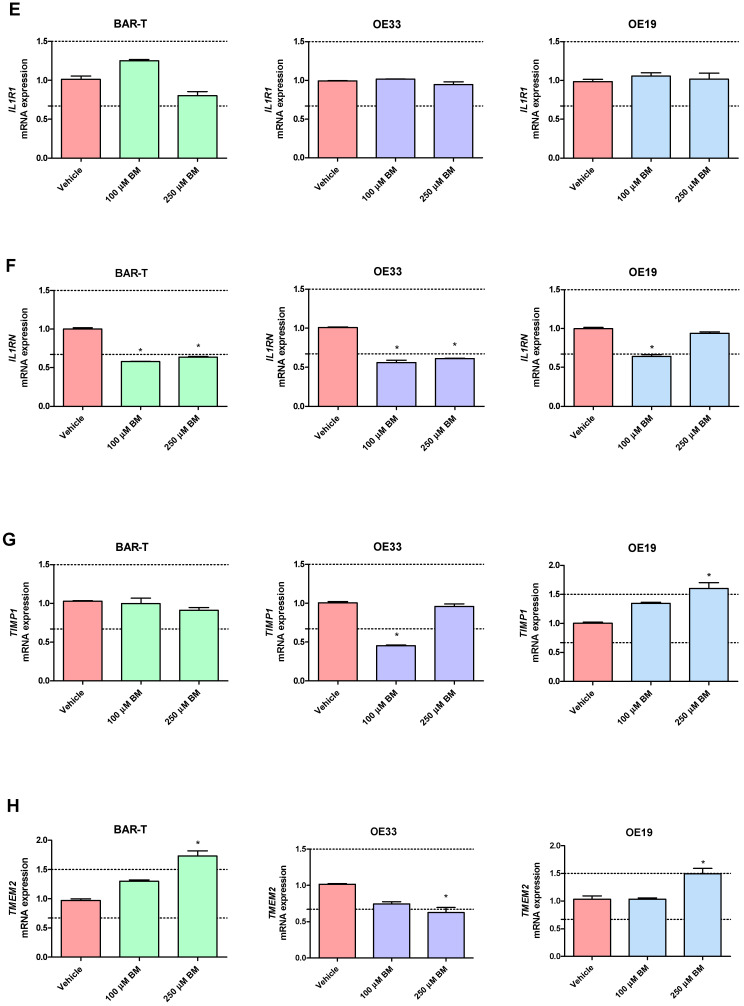

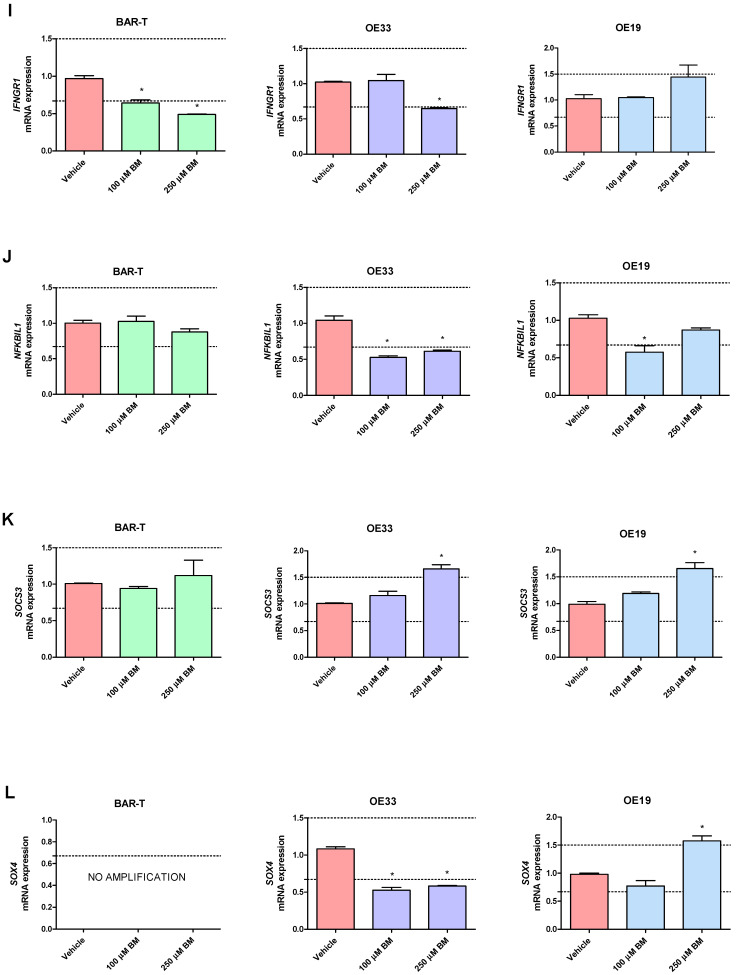

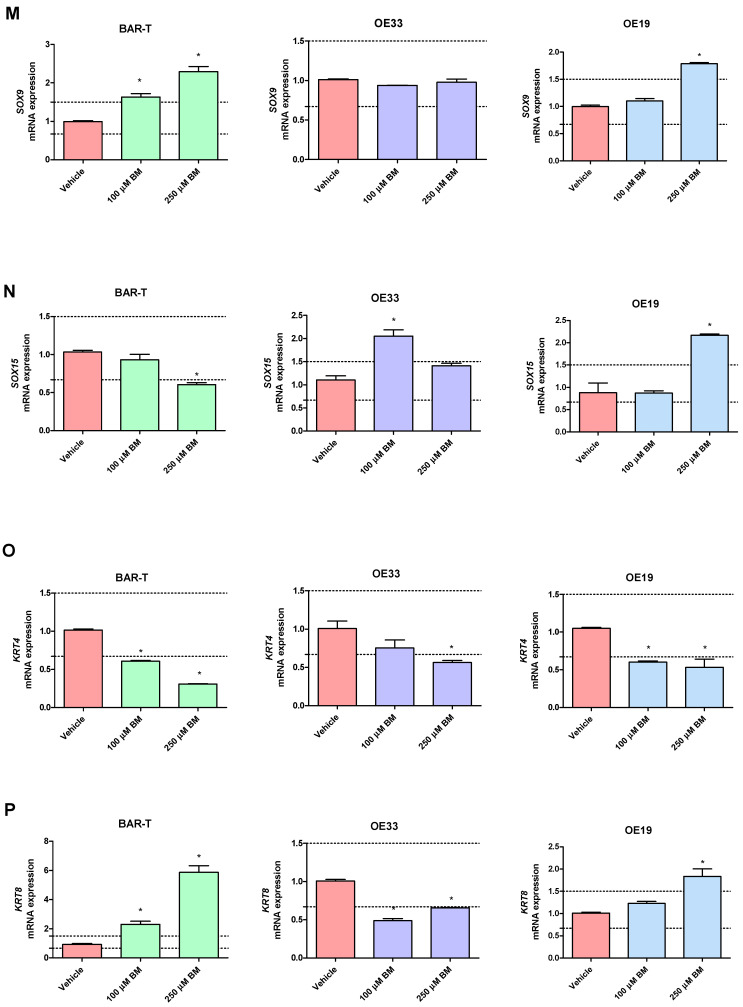

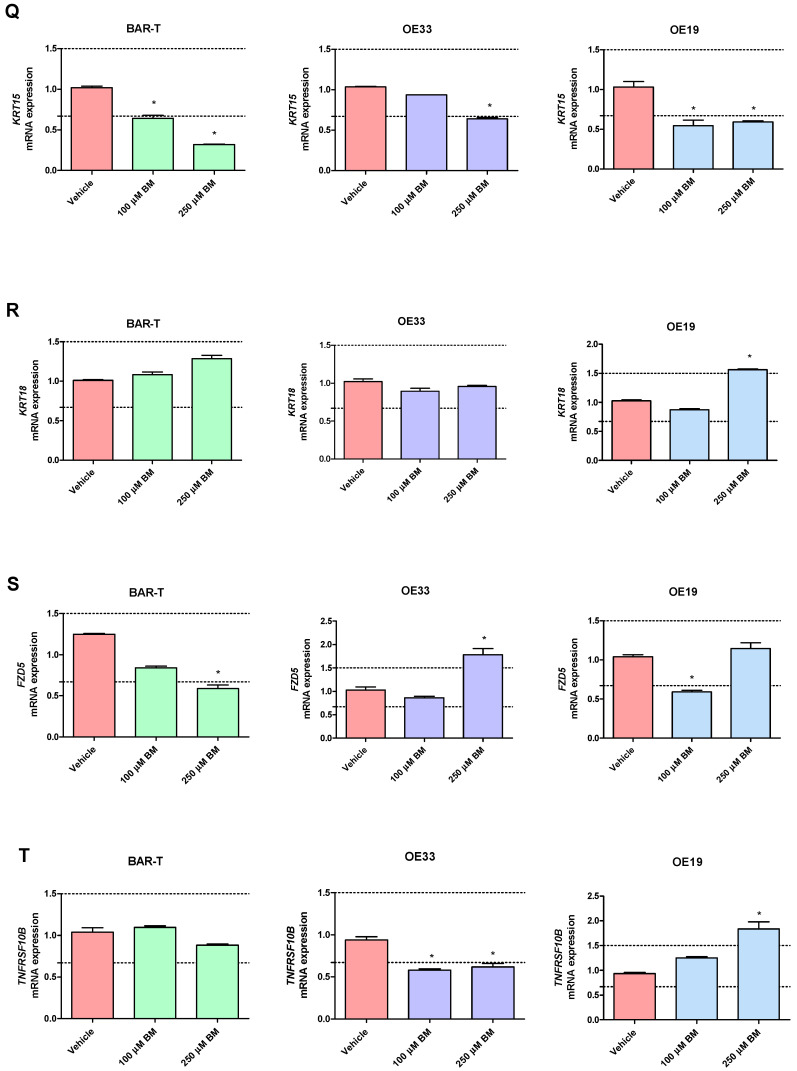
Alterations in *PTGS1* (**A**), *PTGS2* (**B**), *IL1A* (**C**), *IL1B* (**D**), *IL1R1* (**E**), *IL1RN* (**F**), *TIMP1* (**G**), *TMEM2* (**H**), *IFNGR1* (**I**), *NFKBIL1* (**J**), *SOCS3* (**K**), *SOX4* (**L**), *SOX9* (**M**), *SOX15* (**N**), *KRT4* (**O**), *KRT8* (**P**), *KRT15* (**Q**), *KRT18* (**R**), *FZD5* (**S**), *TNFRSF10B* (**T**) mRNA expression profiles for BAR-T, OE33 and OE19 cells treated for 6 days with acidified bile mixture (BM) to reflect clinical gastroesophageal reflux disease (GERD). Cell lines were exposed daily to 100 or 250 μM of BM applied in acidified medium. Vehicle indicates the cells cultured in regular medium without BM. Dotted lines indicate 1.5-fold up-/downregulation of mRNA expression compared to vehicle. Results are shown as the mean ± SEM of 3 values per experimental group. Statistically significant changes compared with a vehicle were marked with an asterisk (*) (*p* < 0.05).

In BAR-T cells treated with 100 μM of BM, mRNA expression of *SOX9* and *KRT8* was significantly increased, and the mRNA expression of *PTGS1, IL1RN, KRT4, KRT15, IFNGR1* was significantly decreased compared to vehicle (*p* < 0.05, Figure 3A–T). The mRNA expression of *PTGS2, IL1A, IL1B, IL1R1, TMEM2, TIMP1, NFKBIL1, SOX15, SOCS3, KRT18, TNFRSF10B* and *FZD5* was not significantly altered in BAR-T cells compared to vehicle (Figure 3A–T). Upon incubation with 250 μM of BM, the mRNA expression of *PTGS2, TMEM2*, *SOX9, KRT8* was significantly upregulated, whereas *PTGS1, IL1RN, SOX15, IFNGR1, KRT4, KRT15, FZD5* were decreased compared to vehicle (*p* < 0.05, Figure 3A–T). *IL1A, IL1B, IL1R1, TIMP1, NFKBIL1, SOCS3, KRT18* and *TNFRSF10B* mRNA expression was not significantly altered compared to vehicle (Figure 3A–T). The mRNA expression of *SOX4* was not detectable in BAR-T cells (Figure 3L). BAR-T cells did not survive 6 days of treatment with 500 μM of BM applied in acidified medium. Thus, the mRNA expression was not determined in this experimental group.

In OE33 cells exposed to 100 μM of BM, significantly elevated mRNA expression of *PTGS2* and *SOX9* was observed, while the mRNA expression of *IL1RN, TIMP1, NFKBIL1, KRT8, TNFRSF10B, SOX4* was downregulated compared with untreated control (*p* < 0.05, Figure 3A–T). The mRNA expression of *PTGS1, IL1A, IL1B, IL1R1, TMEM2, IFNGR1, SOCS3, SOX15, KRT4, KRT15, KRT18* and *FZD5* was not significantly altered compared to vehicle (Figure 3A–T). In OE33 cells exposed to 250 μM of BM, significant upregulation of *PTGS2, IL1A, IL1B, SOCS3, FZD5* mRNA fold change and significant downregulation of *IL1RN, TMEM2, IFNGR1, SOX4, NFKBIL1, KRT4, KRT8, KRT15 and TNFRSF10B* compared with untreated control were observed (*p* < 0.05, Figure 3A–T). The mRNA expression of *PTGS1, IL1R1, TIMP1, SOX9, SOX15, KRT18* was not altered compared with vehicle (Figure 3A–T). Microscopically visible morphological changes were observed in the OE33 cells after 500 μM of BM treatment for 6 days. Thus, the mRNA expression was not determined in this experimental group (data not shown).

In OE19 cells treated with 100 μM of BM, significant downregulation of *IL1A, IL1RN, NFKBIL1, KRT4, KRT15* and *FZD5* but not of *PTGS1, PTGS2, IL1B, IL1R1, TIMP1, TMEM2*, *IFNGR1, SOCS3, SOX4, SOX9, SOX15, KRT8, KRT18, TNFRSF10B* mRNA fold change was observed compared with untreated control (*p* < 0.05, Figure 3A–T). OE19 cells exposed to 250 μM of BM were observed to have significantly elevated *PTGS1, IL1B, TIMP1, TMEM2, SOCS3, SOX4, SOX9, SOX15, KRT8, KRT18, TNFRSF10B* and decreased *IL1A, KRT4* and *KRT15* but not *PTGS2, IL1R1, IL1RN, IFNGR1, NFKBIL1* and *FZD5* mRNA expression compared with vehicle (*p* < 0.05, Figure 3A–T). Microscopically visible morphological changes were observed in the OE19 cells after 500 μM of BM treatment for 6 days. Thus, the mRNA expression was not determined in this experimental group (data not shown).

## 3. Discussion

BE is a pre-malignant phase of EAC development. Interactions between the molecular pathways involved in the pathogenesis of this disease are complex and are still not fully investigated. Moreover, pharmacological interventions efficiently affecting BE and EAC progression are limited, leaving a scientific and therapeutic niche to be filled with novel medical technologies. There are several completed clinical trials focused on the effectiveness of well-known drugs used in the chemoprevention of BE and EAC [28,29]. However, taking into account that BE and EAC development is based on long-term and chronic exposure to GERD, these basic and clinical research areas are difficult to handle under experimental conditions [30]. Nevertheless, several in vitro and in vivo animal models of GERD leading to BE were developed [31,32,33]. It was reported that chronic exposure of BE cell lines (BAR-T) to acidified BM (200 μM, pH4) for up to 6 weeks induced an increase in COX-2 expression [31]. Additionally, BAR-T cells exposed daily to acidified BM for up to 65 weeks may confer a transformation of BE cells into a tumorigenic phenotype [32]. The same authors have studied the genetic basis of the metaplasia-dysplasia-carcinoma differentiation and its factors in a BE carcinogenesis model [34]. Upon the treatment of BAR-T cells in similar conditions, they observed BM-induced chromosomal aberrations that led to the development of neoplastic features in BAR-T cells [34]. Moreover, it is important to mention the surgical generation of an esophagogastroduodenal anastomosis in rats or genetic modification leading to overexpression of *IL1B* in mice [35,36]. Additionally, the in vitro models based on human-derived esophageal cell lines have been considered to be poorly adequate and applicable with debatable translational potential. However, this area is advancing and bringing new possibilities for molecular pathophysiology and pharmacology, addressing an urgent need for optimized cellular models to study BE and EAC prevention and/or development with possible implementation in molecular pharmacology. For instance, Nakagawa et al. have reviewed and summarized various validated human cell lines used in BE and EAC research [37].

In our study, we made an attempt to select 20 representative targets previously described to be possibly altered on mRNA expression level or to be involved in the course of GI pathologies such as esophagitis and the development of BE and/or GI cancers (EAC and others) based on scientific literature screening. These markers, but not necessarily pharmacological targets, were put together in four functional groups (Table 3). Moreover, each of the initially selected genes was further verified based on GSE1420 database analysis to confirm or exclude significant expression changes in clinical biopsies derived from patients with diagnosed EAC (Table 1). The selected genes encode proteins that interact with signaling pathways, pleiotropic cytokines, transcriptional or transmebrane proteins involved in cell differentiation, cancer progression or aggressiveness. Acid reflux was supposed to cause inflammation in gastroesophageal mucosa, which contributes to GERD-related carcinogenesis and BE development via the expression of pro-inflammatory cytokines and the activation of NF-κB and other molecular signaling cascades [38,39]. The interleukin 1 (IL1) family pathway with its downstream effectors (*IL1A*, *IL1R1, IL1RN, IL1B*) are esophagus-specific genes and were shown in previous studies to be linked, among others, with the development and progression of acute esophagitis and BE [38,39,40]. Moreover, the overexpression of IL1B in squamocolumnar junctions triggers BE metaplasia/dysplasia to cancer in mouse models [35,41]. IFNGR1 belongs to the interferon-γ receptor family and is engaged in the regulation of the JAK1/2/STAT-1 pathway. Its decreased expression was observed in ESCCs, enhancing the resistance to apoptosis [42]. SOCS3 is a protein related to the downregulation of the JAK/STAT3 signaling pathway, which was shown to be an inhibitory molecule expressed in tissues with chronic inflammation [43]. Additionally, it was demonstrated that the suppression of SOCS3 has an impact on progression from pancreatic intraepithelial neoplasia to pancreatic ductal adenocarcinoma [44]. The NFKBIL-1 protein was shown recently to be a potential biomarker of diabetes-related colorectal carcinoma [45]. FZD5 is a membrane receptor related to Wnt/β-catenin signaling. The activation of Wnt was found to be a key pathway in EAC development in an in vitro BE model [46]. In OE19 cell lines, FZD5 was also reported to be strongly expressed [35]. SOX family members, including SOX4, SOX9, and SOX15, are very often studied in relation to cancer and were observed to be related to the suppression of the Wnt/β-catenin pathway [47]. SOX4 is overexpressed in GI cancers and is associated with poor prognosis [48]. Furthermore, it was demonstrated to exert pro-oncogenic function involving the regulation of the Wnt, TGF-β, PI3K pathways and to interact with key proteins (β-catenin, FZD5, p53, SMAD3, TMEM2) and many others [49]. In esophageal carcinomas, SOX4 promotes tumor progression and invasion [50]. Increased expression of SOX9 was found to drive columnar differentiation in squamous esophageal epithelium MES cells and in the in vivo reconstruction model [51]. A comparative study of mutational patterns has shown that SOX9 is frequently mutated in GI adenocarcinomas [52]. The SOX15 gene was demonstrated to be a tumor-suppressing gene, downregulating the Wnt/β-catenin pathway in esophageal cancer [53]. TMEM2 is regulated by the above-mentioned SOX4 protein and has been shown to promote invasion in breast cancer [54]. It was also suggested to be a poor prognosis factor in pancreatic ductal adenocarcinoma [55]. In our study, we have analyzed the KRT gene family including *KRT4, KRT8, KRT15* and *KRT18*, the epithelial terminal differentiation markers. KRT4 expression is downregulated in leukoplakia and oral squamous cancer carcinomas [56,57]. KRT8 overexpression was connected to gastric cancer cell proliferation and progression in vitro [58]. However, highly expressed KRT15 protein may contribute to esophageal carcinoma progression [59], whereas overexpression of KRT18 in colorectal cancer exerts an oncogenic role [60]. Moreover, our previously published data confirmed that these markers reflect clinical alterations within an in vitro model of BE [21]. The expression of COXs (COX1 and COX2, encoded by *PTGS1* and *PTGS2*) was linked to the development of GI tumors and early BE-derived neoplastic transformation [61,62]. TIMP1 was shown to be upregulated in EAC lesions when tumor cells invade into esophageal mucosa [63]. The cell surface receptor TNFRSF10B (or death receptor 5-DR5), from the tumor necrosis factor family, mediates the extrinsic apoptosis pathway, and its upregulation is associated with BE-related adenocarcinomas [64].

In our analysis, we divided the selected genes into four groups: genes encoding inflammatory response pathways; columnar- or squamous-epithelium specific genes; genes expressed in oncogenic Wnt/β-catenin signaling pathways; and genes that regulate cell proliferation and apoptosis (Table 3).

All these targets, except *IL1R1*, were confirmed in our analysis of the GSE1420 database to be significantly altered in clinical EAC biopsies compared to healthy squamous esophageal epithelium. Therefore, we have further evaluated if mRNA expression of these selected markers is changed in vitro across BE- and EAC-representing cell lines. We also verified possible additional alterations of these genes after chronic exposure of these cell lines to acidified BM, mimicking clinical GERD under experimental conditions. Alterations of these markers’ expression could be used as sensors, but not necessarily targets, to evaluate the effectiveness of possible novel pharmacological interventions on the development of BE and EAC.

We employed a BAR-T cell line representing non-neoplastic cells derived from non-dysplastic BE biopsies and immortalized with human telomerase reverse transcriptase (hTERT) [72]. We also verified our hypothesis assuming significant differences between the molecular profiles of cancer cells isolated from EAC since the cell lines vary in tumor stage and grade. Precisely, TNM (T—tumor, N—nodes, M—metastasis) is a cancer stage evaluating system that classifies the extent of cancer spread, while its grade corresponds to the cancer aggressiveness level (poor, medium or high differentiation of cells). Both parameters are used to describe esophageal cancers [73]. The OE33 cell line, implemented in our study, was isolated from an EAC tumor located in the distal esophagus that indicated a IIA stage (UICC) and showed poor differentiation (G3). It corresponds to the T2N0M0 according to the novel classification of esophageal tumors [74]. The OE19 cell line was established from EAC of the esophageal gastric junction/gastric cardia in stage III (UICC) with moderate differentiation (G2) that corresponded to T2-T3N2M0 [74]. Interestingly, exposure of esophageal dysplastic and EAC cells to BM was suggested previously to be linked with further BE progression and EAC development [25,26,27,31,32,34,39]. In EAC cells (OE33, OE19, FLO-1) exposed to 200 μM of acidified BM for 20 min, enhanced cancer cell survival was noted due to EGFR-DNA-PKcs pathway activation via insulin-like growth factor binding protein 2 (IGPBP2) [75]. Other authors showed that cardiac glycosides efficiently inhibited OE33 and OE19 proliferation in vitro and in vivo; however, the BM effect was not verified in this study [76]. Therefore, we hypothesize that maintained exposure to GERD in patients with EAC might have a negative impact on possible anti-cancer treatment and could still induce additional molecular alterations. We also assumed that a more advanced metaplasia or neoplasia stage increases the resistance to the BM-induced cell death and/or expression pattern. Indeed, we observed that the viability of OE33 and OE19 cells was significantly decreased in pH 5.0 when BM was applied at a concentration of 750 µM and 1000 μM, respectively. In turn, the viability of BAR-T cells was significantly decreased in pH 5.0 when BM was applied at a concentration of 500 μM. These data showed that OE19 cells are more resistant to treatment with high concentrations of acidified BM as compared with OE33. Consequently, OE33 are more resistant than BAR-T cells. We also observed a different gene expression pattern between OE33 and OE19 cell lines (Figure 1F), as discussed in detail below.

Interestingly, no cell inhibition or differences in cell viability were observed after BM treatment at pH 7.0 in all investigated cell lines. This could partially be explained by the fact that lowering pH towards pKa values of unconjugated (pKa 5.2–6.2; e.g., deoxycholic acid) or glycine-conjugated (pKa 3.8–4.8; e.g., sodium glycocholate) may unionize bile salts, which makes them more lipophilic and able to enter the epithelial cells by cell membrane rupture and influence intracellular pathways [24]. This is also in accordance with our previous study, where we observed that acidified BM is more cytotoxic for esophageal HET-1A and EPC2 cells [21].

As mentioned above, in our study, we screened 20 genes that might reflect BE progression to EAC as a consequence of chronic exposure to acidified BM, and we evaluated the clinical molecular profile of these targets by the analysis of patient-derived data from the GSE1420 dataset deposited in the Gene Expression Omnibus (GEO) [77]. Based on this database, Kimchi E.T. et al. studied normal esophageal epithelium progression to EAC via BE in human biopsies. They identified several molecular markers mostly associated with the suppression of epidermal differentiation [77]. Nevertheless, biomarkers involved in the progression of BE have not been fully studied. Recently, Shen et al. aimed to identify novel molecular markers specific for BE and re-analyzed the GSE100843, including BE segment and normal squamous mucosa samples before and after vitamin D3 supplementation [78]. An analysis based on the GSE1420 and GSE26886 datasets was conducted by Lv J et al. to identify potential biomarkers for BE and EAC [45]. A recent work of Zhang Q. et al. determined that exposure of bile salts to non-neoplastic and neoplastic BE cell lines (BAR-T, BAR-10T, BEC-20W, CP-B, CP-C and CP-D) evoked epithelial to mesenchymal transition (EMT) features; however, in non-neoplastic cells, even short exposures of up to 30 min induced EMT on a molecular level [79]. Moreover, the authors claimed that only BE cells treated with acidic bile salts can induce EMT [79].

We observed that 19 out of 20 (95%) selected genes were significantly altered in EAC biopsies vs. normal squamous esophageal epithelium (Table 1). Interestingly, only 6/20 (30%) of these genes were changed in EAC clinical samples compared to BE (Table 1). This includes cyclooxygenase-1 (*PTGS1*), IL1 (*IL1A, IL1RN*), Wnt/β-catenin signaling pathway components (*SOX15*) and columnar epithelium-specific keratins (*KRT4, KRT15*). Moreover, based on the analysis of 3 clinical datasets, we noticed that similarly to EAC, in BE biopsies, 19 out of 20 (95%) selected genes were altered vs. squamous epithelium (Table 2). This shows that the most significant molecular switch is observed in the metaplasia phase, while the discrepancies between metaplasia and neoplasia are not widely expressed. We confirmed this in our in vitro studies where we observed that the basal gene expression profile correlated with the clinically observed alterations between EAC and squamous epithelium for 15 out of 20 genes (75%) in OE33 vs. EPC2 and for 13 genes out of 20 genes (65%) in OE19 vs. EPC2 cells (Table 4). However, the basal gene expression profile for both OE33 and OE19 vs. BAR-T cells reflected only 10 out of 20 genes (50%) altered between clinical EAC and BE (Table 5). We also observed different gene expression patterns for 13 out of 20 (65%) genes between OE33 and OE19 cell lines (Figure 1F). Altogether, this shows that the investigated cell lines not only may reflect the molecular switch observed in human biopsies, but also could indicate different phases of EAC development or even different types of cancer. This is in accordance with Panda et al., who constructed and validated gene expression signatures of EAC vs. ESCC tumors using publicly available datasets. They observed that OE33 clusters are close to primary EAC tumors, while OE19 could be either classified as an EAC or ESCC cell line [80]. To sum up, differences in gene expression patterns require careful evaluation to enable the selection of appropriate in vitro tools for future BE and EAC studies. For instance, Tratnjek et al. have attempted to standardize an EAC in vitro model for drug testing using an esophageal adenocarcinoma FLO-1 cell line in anticancer drug studies and EAC tumor biology [81]; however, according to Panda et al., this cell line may not be suitable for studying EAC tumors [80]. Furthermore, it is worth mentioning that cell cultures apparently lack a mechanical microenvironment and the proper distribution of oxygen, nutrients, and metabolic products; thus, 3D culture models are a novel option used in the research of esophageal diseases as a perspective in future research [82].

Moreover, and importantly, we compared changes in the expression of selected genes observed in BAR-T, OE33 and OE19 cell lines after BM (100, 250 μM) treatment to gene expression altered between EAC and squamous epithelium in clinical biopsies. We excluded from our analysis the dose of 500 μM. Based on a cell viability assay (MTT), we have selected BM at the concentration of 500 µM applied in medium adjusted to pH 5.0 as the concentration which did not affect cell growth. However, this was verified after one exposure to BM (30 min). In the implemented in our study in vitro model of GERD, cells were exposed to BM 500 μM (30 min) for 6 days, and we observed that many cells started to release their cellular cytoplasmic contents into the extracellular space (data not shown). Speculatively, a higher concentration may activate several regulatory mechanisms, resulting in cell death. As expected, we did not observe any changes in cell morphology after the BM treatment at the concentrations of 100 μM and 250 μM. Additionally, GI mucosa is known to adapt after chronic exposure to low doses of noxious agents such as, e.g., acetylsalicylic acid [83,84]. We assumed that the non-linear response seen for the expression of a few molecular targets in cells between groups treated with BM applied in 100 and 250 μM could be due to enhanced adaptation under these specific experimental conditions. A gene expression comparison led us to conclude that the higher dose (250 µM) of BM better reflects (10/19 vs 7/19 for BAR-T, 9/20 vs. 4/20 for OE33 and 13/20 vs. 7/20 for OE19) the pattern observed in human biopsies for BAR-T, OE33 and OE19 cell lines (Table 5). Interestingly, we also observed that in OE19 cells, BM treatment in a higher percentage reflected alternations in gene expression between clinical EAC and squamous epithelium. Nevertheless, it is still difficult to draw unambiguous conclusions regarding the progression status of EAC cell lines. This could be partially due to limitations resulting from the origins of clinical samples that represent various heterogenous histologic EAC types [77]. The presence of well-known mutations reported in genes that commonly occur in EAC, known as driver mutations like *ARID1A, CDKN2A, KRAS, APC, SMAD4,* and *PI3KCA,* and a high tumor mutation burden (TMB) level makes EAC a highly heterogeneous disease [85,86,87].

Interestingly, basal expression in BAR-T vs. EPC2 cells was similar for 14 out of 20 (70%) genes altered in clinical BE vs squamous epithelium (Table S1). Moreover, BAR-T cells within an in vitro model of GERD covered 12 out of 20 (60%) gene patterns observed between clinical EAC and BE, including these without any deregulations, as well as the reflected molecular changes for 13 out of 20 (65%) genes altered within basal expressions in OE33 vs. untreated BAR-T cells and 10 out of 20 (50%) genes altered within basal expressions in OE19 vs. untreated BAR-T (Table 5).

Our study confirmed that within a BAR-T-based in vitro model of GERD, among genes encoding inflammatory response pathways, two genes, *IL1RN* and *PTGS1*, were downregulated, while *PTGS2* was upregulated. Among the *KRT* family, squamous epithelium-specific *KRT4* and *KRT15* were downregulated, and columnar epithelium-specific *KRT8* was upregulated. In the group encoding the Wnt/β-catenin signaling pathway, *SOX9* and *TMEM2* were upregulated, whereas *SOX15* and *FZD5* were downregulated. In the last group related to cell proliferation and apoptosis pathways, only *IFNGR1* was downregulated (Table 5). These observations reflected the molecular pattern observed in clinical samples and suggest that the in vitro BAR-T-based GERD model is appropriate and sufficient to be implemented and considered as a translational model for the evaluation of BE progression into EAC during chronic exposure to BM. This is in accordance with Bajpai et al., who used a comprehensive approach integrating large-scale genomic and epigenomic datasets to identify molecular alterations in the BE carcinogenesis model acquired during 20 weeks of acid and bile exposure. They observed widespread novel changes in the transcriptome, methylome, and mutatome upon comparing the untreated BAR-T cells with those exposed to 20 weeks of acid and bile [88]. There are also several more studies that confirm the link between GI cancers and BM exposure [89]. For instance, Huo et al. demonstrated molecular mechanisms whereby bile reflux might contribute to carcinogenesis in Barrett’s esophagus. Their data show that in Barrett’s epithelial cells, bile acids induce ROS production, which activates the NF-κB pathway and enables cells to resist apoptosis [90]. In turn, Xu et al. developed a 1-year exposure model of preneoplastic Barrett’s cells to oncogenic bile acid and examined the hypothesis that minority mitochondrial outer membrane permeabilization facilitates cellular transformation and carcinogenesis while promoting resistance to apoptosis [91]. Our results reported in this manuscript confirmed previously published findings indicating that exposure to acidified BM contributes to carcinogenesis in non-dysplastic BAR-T cells.

## 4. Materials and Methods

### 4.1. Evaluation of EAC and BE Gene Expression Profiles in Clinical Samples Based on the GSE Database

The GSE1420 database [77] covering patient-derived biopsies of normal esophageal epithelium (8 samples), BE metaplasia (8 samples) and EAC (8 samples) [21,77] was analyzed in silico using the NCBI Gene Expression Omnibus (GEO) and the GEO2R bioinformatic tool (www.ncbi.nlm.nih.gov/geo/geo2r/, accessed on 1 February 2021), as described previously [21]. Results were reported as a log2-fold change (logFC) in EAC vs. normal epithelium samples and EAC vs. BE samples. The software calculations using the empirical Bayes statistics, moderated for each logFC, reflected the possible differences in gene expression. Statistical significance was considered for the data with *p* < 0.05. The genes with logFC values >1 were considered to be significantly upregulated, and logFc values < −1 were considered to be downregulated. To evaluate the progression of BE and EAC within the in vitro models described below, 20 genes were selected (*SOCS3, SOX4, SOX9, SOX15, FZD5, KRT4, KRT8, KRT15, KRT18, PTGS1, PTGS2, TMEM2, IFNGR1, TIMP1, TNFRSF10B, IL1B, IL1R1, IL1RN, IL1A, NFKBIL1*) as possible molecular markers of BE and EAC development based on a scientific literature screening, described in details in the Discussion section [38,39,40,41,42,43,44,45,46,47,48,49,50,51,52,53,54,56,57,58,59,60,61,62,63,64,65,66,67,68,69,70,71,92,93,94].

To evaluate the alterations of the selected genes at each pathological stage, alterations within the above-mentioned molecular markers in BE biopsies vs. squamous epithelium were also assessed. For this purpose, GSE1420 [77], GSE34619 [92], and GSE13083 [93] were analyzed as described in detail above.

### 4.2. Cell Cultures

The human-derived EAC cell lines OE33 and OE19 and a human Barrett’s metaplasia-derived BAR-T cell line were implemented for in vitro experiments [94]. The OE33 cell line was established from an adenocarcinoma of the lower oesophagus in stage IIA (UICC) [95]. The OE19 cell line was derived from an adenocarcinoma of the gastric cardia/oesophageal gastric junction in stage III (UICC) [95]. Additionally, primary human-telomerase reverse transcriptase (h-TERT) immortalized EPC2 derived from squamous epithelium was used as a reference [96,97]. The OE33 and OE19 cell lines were a gift of Dr. Ron Smits and Dr. Winand Dinjens (Erasmus MC, Rotterdam, The Netherlands). The BAR-T cell line was a gift of Dr. Rhonda Souza (Baylor Scott & White Research Institute, Dallas, TX, USA). The EPC2 cell line was a gift of Dr. Vincent Janmaat (Erasmus MC, Rotterdam, The Netherlands). OE33 and OE19 cells were cultured in RPMI-1640 medium (Sigma-Aldrich, St. Louis, MO, USA) with 5% fetal bovine serum (FBS, Sigma-Aldrich), 100 U/mL penicillin and 100 μg/mL streptomycin (Sigma-Aldrich). BAR-T cells were maintained in keratinocyte basal medium 2 (PromoCell GmbH, Heidelberg, Germany) supplemented with 5% FBS (Sigma-Aldrich), 100 U/mL penicillin and 100 μg/mL streptomycin (Sigma-Aldrich), 400 ng/mL hydrocortisone (Sigma-Aldrich), 20 ng/mL of human recombinant epidermal growth factor (EGF, Gibco, Life Technologies, Paisley, UK), 70 μg/mL of bovine pituitary extract (BPE, EMD Millipore Corporation, Billerica, MA, USA), 5 μg/mL of insulin (Sigma Aldrich), 0,1 nM of cholera toxin (Sigma Aldrich), 20 μg/mL of adenine (Sigma-Aldrich) and 5 μg/mL of transferrin (Sigma Aldrich).

EPC2 cells were cultured in keratinocyte-SFM medium (Gibco, Life Technologies, Grand Island, NY, USA) enriched with 0.2 ng/mL of EGF (Gibco, Life Technologies), 50 μg/mL of BPE (Gibco, Life Technologies) 100 U/mL penicillin and 100 μg/mL streptomycin (Sigma-Aldrich). All cell lines were maintained in humidified 5% CO_2_ air at 37 °C. Before reaching 80% confluence, cells were subcultured using 0.25% trypsin-EDTA solution (Sigma-Aldrich).

### 4.3. Cell Viability Assay

Cell viability was evaluated by colorimetric assay using thiazolyl blue tetrazolium bromide (MTT, Sigma-Aldrich) dye. Cells were seeded in 6 replicates (2 × 10^4^ cells/well; 100 μL) in 96-well plates and incubated overnight in humidified 5% CO_2_ air at 37 °C. The BM was tested in the concentration range of 50–1250 μM. The BM dilutions were prepared in neutral (pH 7.0) and acidified (pH 5.0) growth media and were added to cells for 30 min. After BM exposure, the cells were washed with phosphate-buffered saline (PBS, PromoCell GmbH). Untreated cells in regular media served as a control. After 24 h, 50 μL of MTT solution per well was added, and the plate was incubated for 4 h in humified 5% CO_2_ air at 37 °C. Subsequently, the MTT solution was removed, and 75 μL of dimethyl sulfoxide (DMSO, Sigma-Aldrich) was added to each well. The intensity of color was measured at 550 nm in a multi-well spectrophotometer (Tecan Sunrise, Mannedorf, Switzerland).

### 4.4. Experimental Model of GERD Induced by Chronic Treatment with BM

The BM treatment was performed to reflect clinical GERD based on the model described previously [21]. Briefly, the BM consisted of 25% deoxycholic acid, 45% sodium glycocholate hydrate and 30% sodium taurochenodeoxycholate (Sigma-Aldrich) and was prepared in appropriate, acidified media (pH adjusted to 5.0 with 7.2 M HCl). The OE33 and OE19 cells were seeded at approximately 10% confluence, corresponding to a density of 1.2 × 10^5^ cells/well on a 6-well plate. The BAR-T cells were seeded at a confluence of 30%, corresponding to a density of 3.6 × 10^5^ cells/well on a 6-well plate. The cell lines BAR-T, OE33 and OE19 were exposed to BM for 6 days with 2 days of recovery and subculturing on the 3rd day. The final concentration of BM for a single 30-min exposure was set to 100, 250 and 500 μM. BM concentrations were selected based on cell viability data. After exposure to BM, cells were washed with PBS (PromoCell GmbH), and regular medium was added. After the last treatment, cells were left for 24 h for recovery. Next, RNA was extracted and stored at −80 °C until further analysis.

### 4.5. Determination of Molecular Profile by Real-Time PCR

The total RNA was isolated using spin columns (Universal RNA/miRNA Purification Kit, EURx, Gdansk, Poland) according to the manufacturer’s manual. The RNA concentration was measured by a NanoDrop One spectrophotometer (ThermoFisher Scientific, Waltham, MA, USA). The reverse transcription reaction was performed using a high-capacity cDNA reverse transcription kit (Applied Biosystems, Foster City, CA, USA). A fold change of mRNA expression was determined by real-time PCR using Quant Studio 3 (Applied Biosystems). The reactions were carried out in a 96-well plate in technical triplicates using 2× TaqMan fast advanced master mix (Thermo Fisher Scientific, Vilnius, Lithuania) and 20× TaqMan gene expression assays (Thermo Fisher Scientific). The list of selected human genes (and reference genes) and the corresponding TaqMan assays was presented in the Supplementary Table S2. The PCR temperature cycling conditions were as follows: 1. Initial incubation—2 min at 50 °C, 2. Denaturation—2 min at 95 °C, 3. 40 cycles—1 s at 95 °C and 20 s at 60 °C per 1 cycle. The data were analyzed using the 2^−ΔΔCt^ method with cDNA derived from untreated cells as a reference sample.

The mRNA expression of the *KRT8* gene was not detectable in EPC2 cells, whereas mRNA expression of *SOX4* was not detectable in BAR-T cells. Therefore, a cycle threshold (C_t_) of 40 (C_t_ = 40) was taken for the calculations. The statistically significant *p*-value in relative gene expression was determined at 0.05. A 1.5-fold change with *p* < 0.05 in relative gene expression was considered to be a biologically relevant value.

### 4.6. Statistical Analysis

GraphPad Prism 5 (GraphPad Prism Software Inc., San Diego, CA, USA) was used to prepare statistical analyses and figures. Unpaired Student’s *t*-test with Welch’s correction or Mann–Whitney U test was performed when two experimental groups were compared, unless otherwise stated. ANOVA with Dunnett’s post hoc test was used when more than two experimental groups were compared. The results were presented as mean ± SEM. The level of significance was set as *p* < 0.05 for all statistical analyses.

## 5. Conclusions

Our study confirmed that the combined analysis of BAR-T, OE33 and OE19 cell lines treated or not with selected concentrations of BM reflects clinical molecular alterations corresponding with the appropriate stage of BE and EAC advancement. This model reflects the conditions in which BE may progress and shows that the optimized concentration of BM possibly trigger tumorigenesis in non-dysplastic esophageal cells. The clinically observed detrimental effect of BM covers the enhanced molecular alternations in a BAR-T-based GERD model. We realize that the most clinically relevant and translational data could be obtained when in vitro models of esophageal disorders are combined with in vivo animal models. Nevertheless, we assume that our data indicates and confirms that in vitro model combining BAR-T and EAC cells exposed throughout an appropriate time period to selected concentrations of acidified BM might be used in further research. This could possibly include the research protocols aiming e.g., to evaluate molecular responses to novel compounds and prodrugs targeting GERD and BE prevention or treatment.

## Figures and Tables

**Table 1:** Alterations in the expression of selected genes in human biopsies derived from patients with esophageal adenocarcinoma (EAC) compared to normal squamous epithelium or to Barrett’s esophagus (BE), based on an analysis of database no. GSE1420. Asterisk (*) indicates statistically significant differences with *p* < 0.05 in parallel with biologically significant logFC values lower than −1 or higher than 1. An arrow up (↑) indicates upregulation of mRNA expression; an arrow down (↓) indicates downregulation of mRNA expression, and a double-sided arrow (↔) indicates no changes in mRNA expression.

Database No. GSE1420 Tissue Type: Adenocarcinoma (EAC)		Adenocarcinoma (*n* = 8) vs. Normal Esophageal Epithelium (*n* = 8)		Log Fc > 1 Log Fc < −1	Adenocarcinoma (*n* = 8) vs. Barrett’s Esophagus (*n* = 8)		Log Fc > 1 Log Fc < −1
Gene Symbol	Gene ID	*p*-Value	logFc		*p*-Value	logFc	
*FZD5*	206136_at	8.69 × 10^−4^ *	1.8969691	↑	0.1408974	0.84570984	↔
*IFNGR1*	202727_s_at	4.46 × 10^−^^4^ *	1.0490922	↑	0.2911745	0.34897639	↔
*IL1A*	20118_s_at	4.37 × 10^−3^ *	−2.1008288	↓	0.0007048 *	−2.10433414	↓
*IL1B*	39402_at	4.50 × 10^−2^ *	1.1091709	↑	0.358504	−0.54312669	↔
*IL1R1*	215561_s_at	4.35 × 10^−2^ *	−0.7384266	↔	0.7571835	−0.13554952	↔
*IL1RN*	216244_at	1.43 × 10^−3^ *	−4.8525406	↓	0.0013027 *	−3.99004005	↓
*KRT4*	213240_s_at	1.43 × 10^−3^ *	−5.7005044	↓	0.0001973 *	−6.2440119	↓
*KRT8*	209008_x_at	2.17 × 10^−7^ *	3.1887753	↑	0.3836391	0.50810515	↔
*KRT15*	204734_at	2.24 ×10^−3^ *	−5.72458	↓	0.0002355 *	−5.39716256	↓
*KRT18*	201596_x_at	2.73 × 10^−4^ *	2.2219105	↑	0.3513037	0.29363309	↔
*NFKBIL1*	209973_at	1.80 × 10^−3^ *	−1.3808285	↓	0.4237075	−0.31419507	↔
*PTGS* *1*	205127_at	5.19 × 10^−3^ *	−1.8055694	↓	0.0407174 *	−1.06765124	↓
*PTGS2*	204748_at	5.04 × 10^−2^ *	1.4108709	↑	0.7493048	−0.25959637	↔
*SOCS3*	206359_at	2.16 × 10^−^^4^ *	2.5970676	↑	0.9710285	−0.01769524	↔
*SOX4*	201416_at	1.96 × 10^−4^ *	1.6060934	↑	0.009393 *	0.77714721	↔
*SOX9*	202935_s_at	8.77 × 10^−4^ *	2.3750816	↑	0.2308111	0.67648591	↔
*SOX15*	206122_at	6.63 × 10^−5^ *	−3.6497616	↓	0.0087003 *	−2.52633017	↓
*TIMP1*	201666_at	3.71 × 10^−^^5^ *	2.4187493	↑	0.0535691	0.85285424	↔
*TMEM2*	218113_at	7.98 × 10^−^^6^ *	2.0502962	↑	0.6940281	0.11423942	↔
*TNFRSF10B*	209294_x_at	2.17 × 10^−3^ *	1.9376771	↑	0.4609293	0.32614131	↔

**Table 2:** Alterations in the expression of selected genes as seen in biopsies derived from patients with Barrett’s esophagus (BE), compared to normal squamous epithelium (without BE). The analysis was performed in three different databases (GSE1420, GSE34619 and GSE13083). Asterisk (*) indicates statistically significant difference with *p* < 0.05 in parallel with biologically significant logFC values lower than −1 or higher than 1. The arrows indicate changes in mRNA expression in BE: an arrow up (↑) indicates upregulation of mRNA expression, an arrow down (↓) indicates downregulation of mRNA expression, and a double-sided arrow (↔) indicates lack of changes in mRNA expression.

Gene Symbol	Gene ID	Database No. GSE1420 (*n* = 16) Target Tissue: Barrett’s Esophagus			Database No. GSE13083 (*n* = 14) Target Tissue: Barrett’s Esophagus			Database No. GSE34619 (*n* = 18) Target Tissue: Barrett’s Esophagus			
		Barrett’s Esophagus (n = 8) vs. Normal Esophageal Epithelium (n = 8)		Log Fc > 1Log Fc < −1	Barrett’s Esophagus (n = 7) vs. Normal Esophageal Epithelium (n = 7)		Log Fc > 1Log Fc < −1	Barrett’s Esophagus (n = 10) vs. Normal Esophageal Epithelium (n = 8)			Log Fc > 1 Log Fc < −1
		*p*-Value	logFc		*p*-Value	logFc		Gene ID	*p*-Value	logFc	
*FZD5*	206136_at	1.58 × 10^−2^ *	1.0512592	↑	7.17 × 10^−1^	−0.00823	↔	8058498	7.86 × 10^−11^ *	1.9280655	↑
*IFNGR1*	202727_s_at	2.44 × 10^−2^	0.7001158	↔	2.10 × 10^−5^ *	1.5619957	↑	8129861	6.36 × 10^−2^	0.406583	↔
*IL1A*	210118_s_at	9.96 × 10^−1^	0.0035053	↔	2.59 × 10^−4^ *	−2.7528729	↓	8054712	3.90 × 10^−2^ *	−3.6254277	↓
*IL1B*	39402_at	1.55 × 10^−5^ *	2.9966785	↑	3.31 × 10^−1^	−0.0601271	↔	8054722	1.15× 10^−2^ *	1.0997835	↑
*IL1R1*	215561_s_at	3.77 × 10^−1^	−0.8625006	↔	6.81 × 10^−2^	−0.1481829	↔	8043995	1.69× 10^−1^	−0.2684032	↔
*IL1RN*	216244_at	9.25 × 10^−2^	−0.6028771	↔	7.82 × 10^−7^ *	−3.95939	↓	8044574	1.65 × 10^−10^ *	−3.9754035	↓
*KRT15*	204734_at	8.15 × 10^−1^	−0.3274174	↔	1.79 × 10^−4^ *	−6.05938	↓	8015337	2.13 × 10^−12^ *	−4.686177	↓
*KRT18*	201596_x_at	1.95 × 10^−3^ *	1.9282774	↑	9.62 × 10^−8^ *	3.4490243	↑	8154725	1.04 × 10^−7^ *	2.024285	↑
*KRT4*	213240_s_at	5.13 × 10^−1^	0.5435075	↔	1.08 × 10^−2^ *	−4.6401743	↓	7963534	1.48 × 10^−6^ *	−5.428193	↓
*KRT8*	209008_x_at	2.25 × 10^−4^ *	2.6806701	↑	4.59 × 10^−12^ *	6.4172871	↑	7963567	1.46 × 10^−13^ *	4.0091987	↑
*NFKBIL1*	209973_at	1.29 × 10^−4^ *	−1.0666335	↓	2.51 × 10^−3^ *	−0.1449743	↔	8118127	7.68 × 10^−1^	−0.0326435	↔
								8177967			
								8179249			
*PTGS* *1*	205127_at	9.30 × 10^−2^	−0.7379182	↔	3.03 × 10^−2^ *	−0.2672243	↔	8157650	1.20 × 10^−6^ *	−1.233037	↓
*PTGS* *2*	204748_at	3.44 × 10^−2^ *	1.6704673	↑	1.55 × 10^−1^	−0.0942814	↔	7922976	1.57 × 10^−1^	0.7297863	↔
*SOCS3*	206359_at	4.66 × 10^−4^ *	2.6147628	↑	1.16 × 10^−1^	−0.2276743	↔	8018864	1.11× 10^−1^	0.2751112	↔
*SOX15*	206122_at	8.35 × 10^−2^	−1.1234314	↓	1.48 × 10^−5^ *	−2.3811471	↓	8012220	3.32 × 10^−10^ *	−1.168799	↓
*SOX4*	201416_at	1.31 × 10^−2^	0.8289462	↔	1.88 × 10^−6^ *	3.2003314	↑	8117165	1.77 × 10^−5^	0.74846	↔
*SOX9*	202935_s_at	3.00 × 10^−3^ *	1.6985957	↑	5.01 × 10^−4^ *	2.4066814	↑	8009517	1.83 × 10^−4^	0.7532475	↔
*TIMP1*	201666_at	1.03 × 10^−2^ *	1.565895	↑	9.31× 10^−8^ *	5.4677657	↑	8167185	2.86× 10^−5^ *	1.8116638	↑
*TMEM2*	218113_at	1.10 × 10^−5^ *	1.9360567	↑	5.36 × 10^−8^ *	3.5432943	↑	8161701	8.05 × 10^−13^ *	3.0865858	↑
*TNFRSF10B*	209294_x_at	2.89 × 10^−3^ *	1.6115358	↑	1.64 × 10^−1^	0.2274829	↔	8149733	1.66 × 10^−11^ *	1.6029245	↑

**Table 3:** Genes selected based on the literature and further evaluated with the GSE1420 database, gathered in main functional groups.

No	Function	Gene
I	Inflammatory response pathways	*IL1A, IL1B, IL1RN, IL1R1, PTGS1, PTGS2, NFKBIL, SOCS3* [38,39,40,41,42,43,44,45,61,62,65,66,67]
II	Epithelial/squamous origin	*KRT* gene family: *KRT4, KRT8, KRT15, KRT18* [56,57,58,59,60,68,69]
III	Wnt/β-catenin signaling pathway	*SOX4, SOX9, SOX15, FZD5, TMEM2* [46,47,48,49,50,51,52,53,54,55,70]
IV	Cell proliferation and/or apoptosis	*IFNGR1,TIMP1, TNFRSF10B* [63,64,71]

**Table 4:** Summarized comparison of gene expression profiles observed between in silico analysis of human esophageal adenocarcinoma (EAC) biopsies vs. normal squamous epithelium and wild type untreated OE33 and OE19 cells normalized to EPC2 cells. An arrow up (**↑**) and blue background indicate significant upregulation; an arrow down (↓) and pink background indicate significant downregulation; and a double-sided arrow (↔) and green background indicate no changes in mRNA expression.

Gene Symbol	Clinical:	Experimental:	Experimental:
	EAC vs. Normal Epithelium	OE33 vs. EPC2	OE19 vs. EPC2
*FZD5*	↑	↑	↑
*IFNGR1*	↑	↓	↓
*IL1A*	↓	↓	↓
*IL1B*	↑	↓	↓
*IL1R1*	↔	↓	↓
*IL1RN*	↓	↓	↓
*KRT15*	↓	↓	↓
*KRT18*	↑	↑	↑
*KRT4*	↓	↓	↓
*KRT8*	↑	↑	↑
*NFKBIL1*	↓	↓	↓
*PTGS* *1*	↓	↓	↓
*PTGS2*	↑	↑	↓
*SOCS3*	↑	↑	↓
*SOX15*	↓	↓	↓
*SOX4*	↑	↑	↑
*SOX9*	↑	↑	↑
*TIMP1*	↑	↔	↔
*TMEM2*	↑	↑	↑
*TNFRSF10B*	↑	↔	↔

**Table 5:** Summarized comparison of gene expression profiles observed between in silico analysis of human Barrett’s esophagus (BE) and esophageal adenocarcinoma (EAC) biopsies, untreated wild type cell lines and BAR-T, OE33 and OE19 cells exposed or not to acidified bile mixture (BM) in an in vitro model of GERD. An arrow up (**↑**) and blue background indicate significant upregulation; an arrow down (↓) and pink background indicate significant downregulation; a double-sided arrow (↔) and green background indicate no changes in mRNA expression; NA—no amplification.

Alternations in Gene Expression—A Summary							
Functional Group	Gene Symbol	Human-Derived EAC vs. BE (GSE1420 Database)	Untreated Wild Type Cell Lines		BAR-T upon 250 μM BM Treatment	OE33 upon 250 μM BM Treatment	OE19 upon 250 μM BM Treatment
			OE33 vs. BAR-T	OE19 vs. BAR-T			
group I	*IL1A*	↓	↓	↓	↔	↑	↓
group I	*IL1B*	↔	↓	↓	↔	↑	↑
	*IL1R1*	↔	↓	↑	↔	↔	↔
	*IL1RN*	↓	↓	↓	↓	↓	↔
	*NFKBIL1*	↔	↔	↔	↔	↓	↔
	*PTGS* *1*	↓	↓	↓	↓	↔	↑
	*PTGS2*	↔	↑	↓	↑	↑	↔
	*SOCS3*	↔	↔	↓	↔	↑	↑
group II	*KRT4*	↓	↓	↓	↓	↓	↓
	*KRT8*	↔	↑	↑	↑	↓	↑
	*KRT 15*	↓	↓	↓	↓	↓	↓
	*KRT18*	↔	↔	↔	↔	↔	↑
group III	*FZD5*	↔	↔	↔	↓	↑	↔
	*SOX4*	↔	↑	↑	NA	↓	↑
	*SOX9*	↔	↑	↑	↑	↔	↑
	*SOX15*	↓	↓	↓	↓	↔	↑
	*TMEM2*	↔	↑	↑	↑	↓	↑
group IV	*TIMP1*	↔	↓	↓	↔	↔	↑
	*TNFRSF10B*	↔	↓	↓	↔	↓	↑
	*IFNGR1*	↔	↓	↔	↓	↓	↔

## Data Availability

All the data supporting the conclusions is included within the manuscript and is available on request from the corresponding author.

## Supplementary Materials

The following are available online at https://www.mdpi.com/article/10.3390/ijms23063312/s1.
