# Supplementary material for: Barrett’s Metaplasia Progression towards Esophageal Adenocarcinoma: An Attempt to Select a Panel of Molecular Sensors and to Reflect Clinical Alterations by Experimental Models
Source: Int J Mol Sci. 2022 Mar 18;23(6):3312. doi: 10.3390/ijms23063312 (PMC8955539; doi:10.3390/ijms23063312)
Supplement: Supplementary file 1 [file ijms-23-03312-s001.zip › ijms-1563370-supplementary.pdf]

Supplementary Materials

Table S1. Summarized comparison of gene expression profiles observed between *in silico* analysis of human Barrett's esophagus (BE) and squamous epithelium biopsies, as well as untreated wild type cell lines EPC-2, BAR-T, OE33 and OE19. Arrow-up (↑**)** and blue background indicate significant upregulation; arrow-down (↓) and pink background indicate significant downregulation; double-sided arrow (↔) and green background indicate no changes of mRNA expression**.**

Gene symbol	Clinical: BE vs squamous epithelium	Experimental: BAR-T vs EPC2	Experimental: OE33 vs OE19
FZD5	↑	↑	↑
IFNGR1	↑	↔	↑
IL1A	↓	↑	↓
IL1B	↑	↑	↓
IL1R1	↔	↓	↑
IL1RN	↓	↓	↔
KRT15	↓	↔	↓
KRT18	↑	↑	↔
KRT4	↓	↔	↑
KRT8	↑	↑	↔
NFKBIL1	↓	↓	↔
PTGS1	↓	↓	↑
PTGS2	↑	↑	↓
SOCS3	↑	↑	↓
SOX15	↓	↓	↔
SOX4	↑	↑	↔
SOX9	↑	↑	↓
TIMP1	↑	↑	↓
TMEM2	↑	↔	↑
TNFRSF10B	↑	↑	↔

Table S2. The list of selected human genes (and reference genes) and the corresponding TaqMan assays.

No.	Gene name and symbol	Assay ID	NCBI gene reference
1.	Glyceraldehyde-3-phosphate dehydrogenase, *GAPDH*	Hs99999905_m1	NM_002046.5
2.	Frizzled class receptor 5, *FZD5*	Hs00361869_g1	NM_003468.3
3.	Interferon gamma receptor 1, *IFNGR1*	Hs00988304_m1	NM_000416.2
4.	Interleukin 1 alpha, *IL-1α*	Hs00174092_m1	NM_000575.4
5.	Interleukin 1 beta, *IL-1β*	Hs01555410_m1	NM_000576.2
6.	Keratin 4, *KRT4*	Hs00361611_m1	NM_002272.3
7.	Keratin 8, *KRT8*	Hs01670053_m1	NM_001256282.1
8.	Keratin 15, *KRT15*	Hs00267035_m1	NM_002275.3
9.	Keratin 18, *KRT18*	Hs02827483_g1	NM_000224.2
10.	Interleukin 1 receptor type 1, *IL1R1*	Hs00991010_m1	NM_000877.3
11.	Interleukin 1 receptor antagonist *IL1RN*	Hs00361869_g1	NM_000577.4
12.	Nuclear factor of Kappa Light Polypeptide gene enhancer in B-cells inhibitor-like protein- 1, *NFKBIL1*	Hs00428211_m1	NM_005007.3
13.	Prostaglandin Endoperoxide Synthase 1 *PTGS1*	Hs00924808_m1	NM_000962.3
14.	Prostaglandin Endoperoxide Synthase 2 *PTGS2*	Hs00153133_m1	NM_000963.3
15.	Suppressor of cytokine signaling 3, *SOCS3*	Hs01000485_g1	NM_003955.4
16.	Sex determinating region Y box- 4, *SOX4*	Hs00268388_s1	NM_003107.2
17.	Sex determinating region Y box- 9, *SOX9*	Hs01001343_g1	NM_000346.3
18.	Sex determinating region Y box- 15, *SOX15*	Hs00199511_m1	NM_006942.1
19.	Tissue Inhibitor Of Metalloproteinases 1, *TIMP1*	Hs99999139_m1	NM_003254.2
20.	Transmembrane protein 2, *TMEM2*	Hs00910523_m1	NM_001135820.1
21.	Tumor Necrosis Factor Receptor Superfamily Member 10B, *TNFRSF10B*	Hs00366278_m1	NM_003842.5
